# Altered Mental Status Due to Amantadine Withdrawal: A Case Report

**DOI:** 10.3390/reports9010085

**Published:** 2026-03-12

**Authors:** Nicole J. Asal, Elisa Piraino, Cristina Hamacher, Husam Abu Nejim

**Affiliations:** 1 Department of Pharmacy Practice and Clinical Research, College of Pharmacy, University of Rhode Island, Kingston, RI 02881, USA; 2 Department of Pharmacy, Brown University Health, Providence, RI 02903, USA; 3 Internal Medicine Residency Program, Brown University, Providence, RI 02903, USA; 4 Geriatrics, Department of Medicine, Loyola Medicine, Maywood, IL 60153, USA

**Keywords:** amantadine, delirium, withdrawal

## Abstract

**Background and Clinical Significance**: Withdrawal symptoms from an abrupt discontinuation or rapid dose reduction in amantadine has been documented as early as 1987. Symptoms can align with several diagnoses, including but not limited to infection, fever, worsening of Parkinson’s disease, seizures, and an altered mental status. In the case described, the timely diagnosis of amantadine withdrawal was delayed due to its nonspecific presentation. **Case Presentation**: A man in his 60s presented with lethargy, confusion, and delayed responses. His past medical history included parkinsonism, a seizure, type 2 diabetes, and schizoaffective disorder. Outpatient medications included amantadine, benztropine, divalproex, levetiracetam, paliperidone, risperidone, and semaglutide. He was admitted for an altered mental status, and home medications were held when he became NPO. A nasogastric tube was placed, and amantadine was restarted. Following the amantadine reinitiation, the patient returned to baseline and, after ruling out other causes, was diagnosed with amantadine withdrawal. He ultimately completed a 20-day admission and was discharged to a nursing home. **Conclusions**: The timely diagnosis of amantadine withdrawal was delayed due to its nonspecific presentation. For patients taking amantadine, clinicians should include amantadine withdrawal in their list of differential diagnoses, and in cases of altered mentation, a careful review of the medication list is essential.

## 1. Introduction and Clinical Significance

Amantadine was initially approved in 1966 for influenza prophylaxis, with its antiparkinsonian effects identified shortly thereafter, and its formal Food and Drug Administration (FDA) approval for Parkinson’s disease was granted in 1973 [1,2]. Although it has been long used to treat motor symptoms in Parkinson’s disease and drug-induced extrapyramidal reactions, its precise mechanism remains unclear. Amantadine is known to act as a weak uncompetitive NMDA receptor antagonist and exhibits both anticholinergic-like and dopaminergic-like effects, contributing to its complex pharmacologic profile [2]. Importantly, clinical concern has grown around amantadine withdrawal syndromes: the abrupt discontinuation or rapid dose reduction can precipitate significant withdrawal symptoms, a phenomenon first described in 1987 and increasingly recognized in contemporary practice [3].

In recently documented cases of amantadine withdrawal, the presentation has been notably nonspecific and mimics other conditions. Symptom trends include fever, tachycardia, an altered mental status, somnolence, and muscular rigidity [4,5,6]. These symptoms can resemble those seen in other well-known conditions like infection, drug toxicity, seizures, dementia, and the progression of Parkinson’s disease, making amantadine withdrawal difficult to recognize. Amantadine withdrawal can be difficult to isolate because the side effects of amantadine can present similarly to withdrawal symptoms (visual hallucinations, confusion, blurred vision, leg edema, dry mouth, and constipation in patients with Parkinson’s disease with levodopa-induced dyskinesias) [2]. This case begins with a nonspecific presentation of what we now know to be amantadine withdrawal symptoms. These symptoms triggered a further investigation into a potential seizure, neuroleptic malignant syndrome (NMS), and aspiration pneumonia, ultimately extending and complicating the patient’s hospital course.

## 2. Case Presentation

A male in his 60s was referred to the emergency department from an outpatient podiatry visit for lethargy, confusion, and slowed responses. The patient was a limited historian at baseline and generally not oriented to date/time. He had a recent mechanical fall without a head strike. Past medical history was notable for parkinsonism (paliperidone-induced), seizure disorder (inactive for 10 years), type 2 diabetes, sleep apnea, and schizoaffective disorder (bipolar type). Outpatient medications at time of admission were notable for amantadine (100 mg by mouth three times daily), benztropine, divalproex ER, levetiracetam, paliperidone (monthly injection), risperidone (as needed), and once-weekly semaglutide. In the emergency department his vitals were stable. Labs were normal with the exception of blood reported on urinalysis, which resolved without intervention. Ammonia was normal, and liver function tests were within normal limits. Both valproic acid and levetiracetam levels were normal (97.13 mcg/mL and <2 mcg/mL, respectively). Initial imaging showed no acute cardiopulmonary process, and computerized tomography (CT) of the head and lungs showed no evidence of acute intracranial bleed or pulmonary embolism, respectively. The patient was admitted for altered mental status. Late on hospital day 2, the patient experienced a choking episode and was made NPO, resulting in the temporary hold of all home medications, including amantadine. On night 3 of admission, the patient became restless and anxious and was yelling out, and 2.5 mg of olanzapine was administered intramuscularly. He was transferred to the ICU for acute mental status changes concerning for neuroleptic malignant syndrome (NMS) related to Parkinson’s. Both neurology and psychiatry were consulted for concern of possible post-ictal state versus pharmacologic effects (olanzapine) or withdrawal (amantadine). Neither specialist had a high suspicion for NMS, citing lack of fever or significant rigidity upon exam. The only symptoms that were consistent with NMS were tachycardia and confusion. Neurology expressed low suspicion of seizure given that the patient was seizure free for 10 years, and previous seizure was due to alcohol withdrawal or lithium toxicity, which the patient was not using or taking at the time of admission. Their recommendations included getting an electroencephalogram (EEG) to rule out seizures, monitoring for infection and other abnormalities via labs, continuing valproic acid and levetiracetam, avoiding further olanzapine or other dopa blocking agents, and resuming amantadine. On day 4, the patient was started on piperacillin/tazobactam for presumed aspiration pneumonia. EEG on day 5 was negative for seizure activity. On day 6, a nasogastric tube was placed due to continued dysphagia, and amantadine was restarted. On day 8, the patient’s alertness and mental status had improved, and he was able to hold a conversation with the medical team for the first time since admission. The patient’s mental status returned to baseline per his family and remained stable for the rest of his admission. Due to persistent dysphagia, home medications were switched to an intravenous route or held. Amantadine continued to be crushed and administered via nasogastric tube. The patient’s hospitalization was further complicated by persistent fevers despite seven days of piperacillin/tazobactam, diarrhea, and dysphagia. He ultimately completed a 20-day admission and was discharged to a nursing home. Divalproex was ultimately switched to liquid valproic acid on day 9 of admission. No other significant medication changes or other diagnoses were made. See Table 1 for a time-anchored summary of events, medication changes, and symptom evolution.

## 3. Discussion

There are several documented cases of amantadine withdrawal in the literature that mirror the patient case presented above. In each presentation, patients were maintained on 200–400 mg of amantadine per day before abrupt discontinuation. The patient in this case was taking 300 mg per day and had been on it for many years, both of which increase the risk for withdrawal. Reasons for withholding amantadine ranged from drug-induced psychosis (from amantadine and other drugs), odynophagia/dysphagia impeding oral intake, and hallucinations concerning for amantadine toxicity [4,5,7,8,9,10]. In these cases, symptoms of withdrawal started three to four days after cessation and included progressive delirium with severe psychomotor hyperactivity, muscle rigidity, fevers, tachycardia, somnolence, and a lack of response to providers [4,5,8,9,11]. Based on the available data, amantadine withdrawal seems to resolve relatively quickly, within 1–2 days after the reinitiation of the drug.

Amantadine facilitates dopaminergic neurotransmission by increasing dopamine release and reducing reuptake in the CNS. An abrupt reduction or discontinuation has been repeatedly shown to precipitate severe withdrawal syndromes—including delirium, catatonia, and neuroleptic malignant syndrome (NMS). Multiple peer-reviewed case series and reports document NMS emerging after amantadine withdrawal, supporting the classification of amantadine withdrawal NMS as a valid subtype within malignant syndromes. Mechanistically, reducing or stopping amantadine acutely lowers dopaminergic signaling and—given amantadine’s additional NMDA-antagonist actions—can unmask a dopamine–acetylcholine imbalance that underlies NMS-like and catatonic presentations; case series further describe a “cortical and limbic dopamine shortage” that reverses rapidly when amantadine is reintroduced [12,13].

Beyond abrupt discontinuation, a dose reduction in amantadine can also provoke withdrawal-related deterioration by acutely lowering the dopaminergic tone. Regulatory labeling for extended-release amantadine warns that rapid dose reduction or withdrawal may cause adverse reactions and recommends tapering (e.g., reducing the dose by half during the final week when discontinuing after more than four weeks of therapy), explicitly advising clinicians to avoid sudden discontinuation because of withdrawal-emergent hyperpyrexia and confusion. Consistent with this, the methodology from the AMANDYSK randomized withdrawal program used a progressive dose replacement over several days specifically to avoid “brutal” withdrawal, acknowledging the risk of hyperthermia-like reactions with more aggressive dose reductions [14,15].

The differential diagnosis for amantadine withdrawal includes multifactorial delirium (from infection, metabolic derangements, or polypharmacy), anticholinergic toxicity from concurrent medications, dopaminergic withdrawal syndrome (parkinsonism-hyperpyrexia syndrome), other drug withdrawal syndromes (e.g., benzodiazepines), and acute encephalitis. Life-threatening conditions that must not be missed include neuroleptic malignant syndrome, serotonin syndrome, and malignant hyperthermia.

While there are still some unanswered questions in this case, such as what explains the patient’s initial symptoms or why the patient developed fevers despite several broad-spectrum antibiotics with no other symptoms of infection, each of these differential diagnoses were ruled out for this patient through various investigations. His symptoms at presentation (confusion, lethargy, and slowed response) were nonspecific. Multifactorial delirium was a consideration, but ultimately metabolic derangements were ruled out along with polypharmacy. While the patient was ultimately treated for presumed aspiration pneumonia, this was not a confirmed diagnosis. The patient showed little to no significant improvement after three days on antibiotics for presumed aspiration pneumonia, but rather his rapid improvement within 24 h of restarting amantadine is more suggestive of amantadine withdrawal rather than infection. Anticholinergic toxicity was ruled out because there were no known changes to his medications that would have increased the anticholinergic burden. Amantadine is primarily excreted by glomerular filtration and tubular secretion, and therefore declines in renal function require a reduction in the dosing frequency [2]. The pH of the urine has been reported to influence the excretion rate of amantadine, and increased monitoring should be employed in circumstances where the urine pH may be affected [2]. The patient’s renal function and pH were stable throughout admission, and he did not have any new amantadine-associated side effects, such as blurred vision, leg edema, dry mouth, or constipation, ruling out amantadine accumulation or toxicity. The lack of fever or significant rigidity upon exam at the time of decompensation ruled out several causes: dopaminergic withdrawal syndromes such as parkinsonism-hyperpyrexia syndrome and neuroleptic malignant syndrome, serotonin syndrome, and malignant hyperthermia. Though there was a low suspicion of seizure (based on the 10-year seizure-free history and the current use of two antiepileptic agents), an EEG was used to definitively rule out seizures.

While there is a well-established collection of case reports that seem to document the symptoms of withdrawal from amantadine, the impact of amantadine discontinuation is not always pronounced or significant. A 2023 study investigated the impact of the discontinuation then reintroduction of amantadine [16]. In this study, 30 patients who had been taking amantadine (mean dose 293.3 mg/day) for 8 years were instructed to stop taking amantadine and report symptoms of withdrawal. Twenty-four of the 30 patients experienced a worsening of motor and non-motor symptoms within 3.6 days of discontinuation, with the most common including tremor (37.5%), gait disturbances (25%), pain (25%), and general weakness (25%). After restarting amantadine again, 92% (22 patients) had a full recovery. Interestingly, 25% of patients experienced no worsening of symptoms despite the discontinuation of amantadine. An important limitation of this study as it relates to the case report presented above is that patients were evaluated over the phone and within one day of discontinuation, which may not have been enough time to fully appreciate its impact. Similarly, other amantadine discontinuation trials have controlled for “rebound or hyperthermia-like symptoms” that can result from an abrupt withdrawal through a tapered discontinuation over several days [15].

Despite these findings, amantadine discontinuation is most often prompted by mental status changes (e.g., visual hallucinations or confusion) and agitation [6,17]. Amantadine withdrawal produces diverse, nonspecific neuropsychiatric and motor symptoms, and the rapid improvement following the drug re-challenge makes recognition crucial. Clinicians should maintain a high level of suspicion for withdrawal and adopt a gradual taper when dose reduction is required, with close monitoring for fever, rigidity, delirium, autonomic instability, or rising creatine kinase.

## 4. Conclusions

The timely diagnosis of amantadine withdrawal is slowed due to its variable presentation. Patients may exhibit nonspecific symptoms resembling an infection, worsening Parkinson’s disease, an altered mental status, or seizures. In cases of altered mentation, a careful review of the medication list is essential. For patients taking amantadine, clinicians should consider amantadine withdrawal in the differential diagnosis when evaluating fever, tachycardia, an altered mental status, somnolence, and/or muscular rigidity.

## Figures and Tables

**Table 1:** Time-anchored summary of events, medication changes, and symptom evolution.

Timepoint	Key Event/Clinical Decision	Medication Changes	Symptom Evolution/Clinical Picture
Pre- admission	Referred from podiatry to ED	Home regimen included amantadine 100 mg TID, benztropine, divalproex ER, levetiracetam, paliperidone, PRN risperidone, semaglutide	Lethargy, confusion, slowed responses
HD 0–1	Admitted	No key changes noted yet	AMS persists; no clear alternative etiology identified
Late HD 2	Choking → NPO	All home meds held, including amantadine stopped abruptly	Sets stage for withdrawal window
Night HD 3	Agitation and ICU transfer; NMS/seizure workup	Olanzapine 2.5 mg IM once; consults advised avoidance of more dopamine blockers and to resume amantadine	Restlessness, anxiety, yelling, tachycardia/confusion noted; NMS less likely due to lack of fever/rigidity
HD 4	Treated as aspiration pneumonia	Piperacillin/tazobactam started	No major improvement attributed to antibiotics early on
HD 5	EEG performed	-	EEG negative, lowering seizure likelihood
HD 6	NG tube placed; amantadine restarted	**Amantadine restarted via NG tube**	Start of rapid recovery phase
HD 8	Marked improvement	Continued amantadine	Mental status improved; could converse; back to baseline per family and stable thereafter
HD 9	Formulation switch for valproate	Divalproex ER → liquid valproic acid	No new primary neurologic decline described
HD 10–20	Ongoing hospital complications	Many home meds IV/held due to dysphagia; **amantadine continued via NG**	Persistent dysphagia, fevers despite antibiotics, diarrhea; discharged after 20 days

HD: hospital day; ED: emergency department; TID: three times daily; ER: extended release; PRN: as needed; AMS: altered mental status; NPO: nothing by mouth; ICU: intensive care unit; IM: intramuscular; NMS: neuroleptic malignant syndrome; EEG: electroencephalogram; and NG: nasogastric.

## Data Availability

The original data presented in the study are included in the article, further inquiries can be directed to the corresponding author.
